# Vaccination status and main contributors to low vaccination coverage in five health regions in the state of Sergipe

**DOI:** 10.1590/1984-0462/2025/43/2024298

**Published:** 2025-12-15

**Authors:** Ariel Oliveira Celestinoa, Thayane Santos Siqueira, Marcelli de Lima Silva, Lucas Maxmyllun Lima Silva, Vinicius Santos Silva, José Rodrigo Santos Silva, Victor Santana Santos, José Roberto Lapa e Silva, Ricardo Queiroz Gurgel

**Affiliations:** a Universidade Federal de Sergipe, Aracaju, SE, Brazil.

**Keywords:** Vaccines, Vaccination coverage, Public health, Vacinas, Cobertura vacinal, Saúde pública

## Abstract

**Objective:**

To measure the percentages of basic pediatric vaccination coverage, of vaccination incompleteness, and to identify their contributors in health regions of the state of Sergipe.

**Methods:**

This is a cross-sectional epidemiological study, carried out through home visits to live births (from 2015 to 2021) for data collection, application of a questionnaire and photographic record of the vaccination booklet in health management regions of Sergipe.

**Results:**

It was possible to show that health regions present heterogeneity in vaccination coverage, as well as vaccination failure in subsequent doses of multi-dose vaccination schedules. Considering the percentages of vaccination incompleteness and the odds ratios, the health region of Propriá, followed by Estância and Lagarto, had the best percentages. Regarding contributors, in the logistic regression analysis, variables such as fear of a reaction and whether or not a friend/relative advised vaccination were highlighted, considering their significant p-value in the regression analysis.

**Conclusions:**

The study provides new results regarding the heterogeneity in vaccination coverage percentages and their contributors in health regions of the state of Sergipe.

## RESUMO

**Objetivo:**

Mensurar os percentuais de cobertura vacinal básica pediátrica, os de incompletude vacinal e identificar seus contribuintes em regiões de saúde do estado de Sergipe.

**Métodos:**

Trata-se de um estudo epidemiológico transversal, realizado por meio de visitas domiciliares com nascidos vivos (de 2015 a 2021) para coleta de dados, aplicação de questionário e registro fotográfico da caderneta de vacinação em regiões de gestão de saúde de Sergipe.

**Resultados:**

Foi possível notar que regiões de saúde apresentam heterogeneidade nas coberturas vacinais, bem como falha vacinal em doses subsequentes de esquemas vacinais multidoses. Considerando-se os percentuais de incompletude vacinal e as razões de chances, a região de saúde de Propriá, seguida por Estância e Lagarto, teve os melhores índices. Com relação aos contribuintes, na análise de regressão logística, variáveis como medo de reação e amigo/parente orientar ou não a vacinar ficaram destacadas, considerando-se seu p-valor significativo na análise de regressão.

**Conclusões:**

O estudo fornece evidências quanto à heterogeneidade dos percentuais de cobertura vacinal e seus contribuintes em regiões de saúde do estado de Sergipe.

## INTRODUCTION

Addressing the decline in vaccination coverage percentages is a global challenge.^1^ In Brazil, the National Immunization Program (NIP), internationally known for its success, has historically maintained its vaccination coverage percentages above 95% since the 1990s.^2^ In recent years, around 2016, the PNI has been facing a decline in these percentages, especially for pediatric vaccines.^1,2,3^

This decline has become a challenge, since the factors that contribute to refusal or delay in vaccination seem to change over time and locality. These factors are heterogeneous, and anti-vaccine movements as well as the dissemination of false information (fake news) have also gained strength in recent times.^2,3,4^

The benefits of widespread vaccination are historically documented in the literature, including herd immunity, a positive economic impact on disease prevention, reduced use of antimicrobials, reduced incidence of vaccine-preventable diseases along with their complications and even deaths.^5,6^

Despite this, the problem of low vaccination rates persists, and there is a clear need to conduct an epidemiological study to measure vaccine coverage percentages in the first two years of life, as well as to identify the main contributors to delay in vaccination in regions of the state of Sergipe, generating scientific data to help public health to design strategies that may restore the vaccination coverage levels seen in the past.^7,8,9^

## METHOD

The study was approved by the ethics committee of the Federal University of Sergipe (UFS), through Certificate of Presentation for Ethical Appreciation — CAAE 42941421.4.0000.5546 and opinion 4669502. Those responsible for the children (born from 2015 to 2021) were asked to sign the Free and Informed Consent Form (TCLE) to consent to their child’s participation. After that, a questionnaire was applied and the child’s vaccination record was photographed, always ensuring the anonymization of personal data, through coding (abbreviation of names) of each participant considering the ethical protection of data.^7,8 9,10^

This is a cross-sectional observational study, conducted through an active search for residences that had live births from 2015 to 2021. The state of Sergipe is located in the northeast region of Brazil, between the states of Bahia and Alagoas, has approximately 21,925.4 km^2^ of extension, and 75 municipalities organized into health regions (Aracaju, Nossa Senhora do Socorro, Estância, Propriá, Lagarto, Itabaiana and Nossa Senhora da Glória).^10^ According to the Brazilian Institute of Geography and Statistics (IBGE), it is estimated that there will be 2,18,822 inhabitants in 2020.^11^

The minimum sample size was estimated considering an infinite population (>10,000), considering a margin of error of 5%, a confidence level of 95% and a significance level of 5%, based on the proportion of 50% of the population results. The minimum sample size was calculated for an infinite population between one and seven years old, resulting in the minimum number of 541 children.^11,12,13^

The inclusion criteria were as follows: children born between 2015 and 2021 (those born in 2021 must be one year old on the date of the visit), who had the vaccination record available on-site (at home), had the TCLE signed by one of their respective guardians, had their guardian answer the questionnaire and allow the photographic recording of the vaccination record.^7^

The variables collected and used were: whether the child attended or had attended a nursery/daycare center; type of delivery; whether the family was a beneficiary of the Bolsa Família/Auxílio Brasil programs; the mother’s level of education; whether the child had been vaccinated through the Brazilian Unified Health System (SUS); whether the child had decided to get vaccinated; whether the child believed that vaccines are harmful; whether the child was afraid of vaccine reactions; whether the news had influenced their decision to skip vaccination; whether a doctor, friend or relative had advised them to get vaccinated; whether lack of transportation, money, vaccines or a professional had been or was a reason for not getting vaccinated.^7^

Regarding the photographic record of the vaccination booklet, the initials of the child’s name, date of birth, health region, type of vaccines and dates of administration of their respective doses were collected, considering vaccines from zero to 24 months of age of the Brazilian Immunization Program (PNI).^7^

The vaccines considered valid for the first and second year of life were evaluated according to PNI guidelines, with the exception of the yellow fever vaccine, which was not collected because it was not recommended for Sergipe at the time.^7,8,9,10,11,12 13,14^

An exploratory analysis was performed using simple frequency and percentage calculations. The percentages of valid doses administered by health regions and by vaccine were graphically represented using a heatmap. Bivariate inferential analysis was conducted using the chi-square and Fisher’s exact tests.^15^ To associate the variables representing compliance with the vaccination schedule with the other study variables, the results were expressed in terms of frequency and calculated by row. The Mann-Whitney test was used to cross-reference the variables with the quantitative variables.^16^ Adherence to a normal distribution was assessed using the Shapiro-Wilk test.^17^

Multivariate analysis was performed using logistic regression adjustment, with a log link function.^18,19^ Variables that presented a p-value lower than 0.20 in the bivariate analysis were included in the regression models.^20^ The area under the Receiver Operating Characteristic (ROC) curve (AUC) was used to measure the quality of the adjustment, and the Hosmer-Lemeshow test was used to assess the goodness of fit. The results were expressed in terms of odds ratio (OR), calculated as the exponential of the estimated parameters.^21,22,23^

The data were organized in Microsoft Excel, and all statistical analyses were performed in R software, version 4.4.0. A significance level of 5% was adopted throughout the study, corresponding to a confidence level of 95%.^24^

## RESULTS

The data were collected in 2022 and 2023 to measure the coverage percentages of vaccines in the first year of life (0–12 months) and the second year (13–24 months). Data from 756 and 648 children were evaluated, respectively.

In the first year, 168 children were included from the Estância region, 175 from Itabaiana, 182 from Lagarto, 119 from Nossa Senhora da Glória, 112 from Propriá. In the second year, 144 were from the Estância region, 150 from Itabaiana, 156 from Lagarto, 102 from Nossa Senhora da Glória and 96 from Propriá.

**Figure 1.**
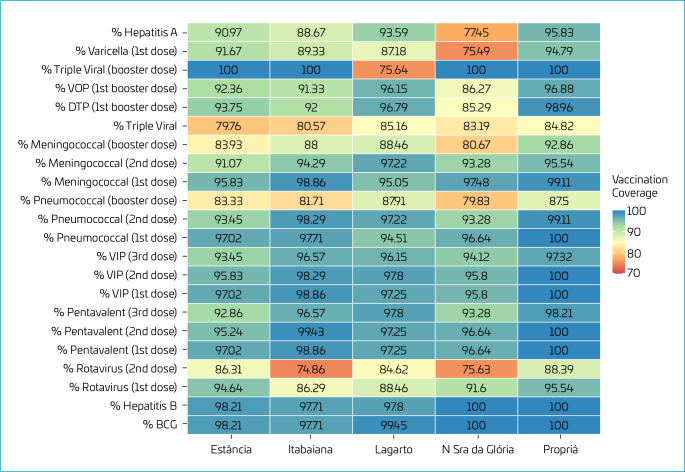
Estimate of vaccination coverage percentage for live births from 2015 to 2021 and residents in health regions of Sergipe.

For the bacillus Calmette-Guérin (BCG) vaccine, the percentages were equal to or greater than 97.71%, with coverage in Propriá and Nossa Senhora da Glória being 100%; in Lagarto, 99.45%; in Estância; 98.21%; and in Itabaiana, 97.71% (Figure 1). For the hepatitis B vaccine, the minimum vaccination coverage was 97.71%. In Propriá and Nossa Senhora da Glória the coverage was 100%, in Estância 98.21%, followed by Lagarto with 97.8% and Itabaiana with 97.71% (Figure 1).

For the first dose of the rotavirus vaccine, the minimum vaccination coverage was 86.29% in Itabaiana. For the second dose, the minimum vaccination coverage rate was 74.86%, also in Itabaiana (Figure 1).

For the pentavalent vaccine, the minimum vaccination coverage percentages were 96.62% in Glória for the first dose, and 95.24 and 92.86% in Estância for the second and third doses, respectively (Figure 1). When considering the percentages of the inactivated polio vaccine (IPV), the minimum coverage percentages were 95.80% for the first and second doses in Glória, and 93.45% for the third dose in Estância (Figure 1).

For pneumococcal vaccine, the minimum rate was 94.51% for the first dose in Lagarto, and 93.28, 79.83% for the second and booster doses, respectively, in Estância (Figure 1). Considering the meningococcal vaccine, the lowest vaccination coverage percentage were in Lagarto, with 95.05% for the first dose, 91.07% for the second dose in Estância, and 80.67% in Glória for the booster dose (Figure 1).

Finally, for vaccines recommended up to 12 months of age, the minimum rate for the MMR vaccine was 79.76%, again in Estância. The other rates were 80.57% for Itabaiana, 83.19% for Nossa Senhora da Glória, 84.82% for Propriá, and 85.16% for Lagarto (Figure 1).

Of the vaccines recommended for children aged 13 to 24 months, the lowest rate overall was 75.49% (Figure 1).

The diphtheria, tetanus and pertussis (DTP) first booster dose vaccine achieved the best vaccination rate in the health region of Propriá with 98.96%, followed by Lagarto with 96.79%, Estância 93.75%, Itabaiana 92% and Nossa Senhora da Glória with 85.29%. As for the oral polio (VOP), first booster dose, the percentage was 96.88% in Propriá, followed by Lagarto with 96.15%, Estância 92.36%, Itabaiana 91.33% and Nossa Senhora da Glória 86.27% (Figure 1).

The triple viral vaccine, recommended with a booster dose at 15 months, reached 100% in the health regions of Estância, Itabaiana, Nossa Senhora da Glória and Propriá. In Lagarto, the percentage was 75.64% (Figure 1). The first dose of the chickenpox vaccine showed a vaccination coverage rate ranging from 94.79% in Propriá to 75.49% in Nossa Senhora da Glória (Figure 1).

Finally, the hepatitis A vaccine percentages were 95.83% in Propriá, 93.59% in Lagarto, 90.97% in Estância, 88.67% in Itabaiana and 77.45% in Nossa Senhora da Glória (Figure 1).

When measuring the percentages of vaccination incompleteness for the vaccines recommended for the first year of life (0 to 12 months), the health region of Itabaiana presented the highest percentage with approximately 31.4% (55), followed by the health regions of Nossa Senhora da Glória with 26.9% (32), Estância with 20.2% (34), Propriá with 16.1% (18) and Lagarto with 12.6% (23) (Table 1).

**Table 1.** Vaccination incompleteness percentages for the first two years of life of children living in regions of Sergipe.

	Incomplete	OR (95%CI)	p-value
0–12 months			
Health regions			
Estância	34 (20.2)	1.3 (0.7–2.5)	
Itabaiana	55 (31.4)	2.4 (1.3–4.3)	
Lagarto	23 (12.6)	0.8 (0.4–1.5)	<0.001
Nossa Senhora da Glória	32 (26.9)	1.9 (1–3.7)	
Propriá	18 (16.1)	1 (ref)	
13–24 months			
Estância	34 (23.6)	2.7 (1.2–5.7)	
Itabaiana	42 (28)	3.3 (1.6–7)	
Lagarto	37 (23.7)	2.7 (1.3–5.7)	0.001
Nossa Senhora da Glória	36 (35.3)	4.7 (2.2–10.1)	
Propriá	10 (10.4)	1 (ref)	
General (0–24 months)			
Estância	41 (28.5)	1.5 (0.8–2.8)	
Itabaiana	71 (47.3)	3.4 (1.9–6.1)	
Lagarto	48 (30.8)	1.7 (0.9–3.1)	<0.001
Nossa Senhora da Glória	43 (42.2)	2.8 (1.5–5.2)	
Propriá	20 (20.8)	1 (ref)	

For vaccines in the second year of life (13–24 months), the highest percentage of vaccination incompleteness was observed in the Nossa Senhora da Glória health region, with 35.3% (36), followed by the regions of Itabaiana with 28% (42), Lagarto 23.7% (37), Estância 23.6% (34), and Propriá, with 10.4% (Table 1).

Overall, for children aged zero to 24 months, the percentage of incomplete vaccination was highest in Itabaiana 47.3% (71) and lowest in Propriá 20.8% (20). The p-value in this age group was significant at 0.000, so that Propriá, followed by Estância, Lagarto, Glória and Itabaiana, had the best percentages (Table 1).

When considering the variables in the questionnaire, of the total of 756 children included in the study: 35.2% (159) of those who did not and had never attended daycare or nursery, 32.1% (62) of those who attended or had attended daycare, 34.5% (38) of the families who reported not receiving government assistance (Bolsa Família/Auxílio Brasil), and 34.1% (173) of the families who did receive it had incomplete vaccinations.

In general (from zero to 24 months), considering variables such as vaccination through the SUS or private sector, the decision to vaccinate the child, and whether or not their guardians believed in vaccines, the percentages of incomplete vaccinations were: approximately 22.2% (2) for children who used the private service and 34.6% (221) for those who used the SUS; 33.9% (211) for children whose guardians had decided to apply all the recommended vaccines; 42.9% (6) for children whose guardians did not believe in vaccines; and 34.2% (216) for those who did believe in vaccines.

Variables such as: opening hours of the Basic Health Unit (UBS), distance from home to the UBS, lack of vaccines, and lack of a professional and closed vaccination room showed, in general, considering the period from zero to 24 months, percentages of vaccination incompleteness of 35.3% (12), 34.5% (38), 40.7% (46), 34.3% (12) and 36.5% (19), respectively, for those who answered yes to these variables as contributing to not vaccinating.

For vaccines in the first year of life, the percentages of vaccine incompleteness were: 26.5% (77) for children whose mothers were categorized as illiterate/completed elementary education (initial years)/incomplete elementary education (final years); 20.5% (35) for those whose mothers had completed elementary education (final years)/incomplete high school; 16.3% (38) for those whose mothers had completed high school/incomplete higher education; and 19.6% (11) for those whose mothers had completed higher education.^25^

For vaccines in the second year of life, the percentages for vaccine incompleteness were: 30.5% (79) for children whose mothers were considered illiterate/having complete elementary school (initial years)/incomplete elementary school (final years); 19.3% (27) for those whose mothers had complete elementary school (final years)/incomplete high school; 20.8% (41) for those whose mothers had complete high school/incomplete higher education; and 21.3% (10) for those whose mothers had complete higher education.^25^

And, for the period from zero to 24 months, the percentages for incomplete vaccination were: 39% (101) of children whose mothers were illiterate/had complete elementary school (initial years)/incomplete elementary school (final years); 32.9% (46) for those whose mothers had completed elementary school (final years)/incomplete high school; 29.4% (58) for those whose mothers had complete high school/incomplete higher education; and 31.9% (15) for those whose mothers had complete higher education.^25^

Regarding the participants who believe that vaccines are harmful, the percentages of incomplete vaccination were approximately 33.3% (6) for the first year, 50% (8) for the second year, and 43.7% (7) considering the period from zero to 24 months.

Among those who expressed fear of a reaction, the rate of incomplete vaccination was 26.9% (78) for the first year, 30.1% (74) for the second year, and 40.7% (100) considering the age range from zero to 24 months. For the variable friend or relative advised to vaccinate, incomplete vaccination was presented by 18.2% (16) of participants regarding first-year vaccines, and 14.5% (12) regarding second-year vaccines.

Considering the lack of transportation and the percentages of incomplete vaccination, the estimate is 33.8% (23) of those who answered yes for the first year, 32.1% (18) for the second year and 42.9% (24%) for the age range from zero to 24 months. For the variable lack of money, the percentages were 41.5% (22) for first-year vaccines, 40% (18) for second-year vaccines and 51.1% (23) considering the period from zero to 24 months.

In the logistic regression, the results (Table 2) indicated that the belief that vaccines are harmful, lack of transportation and lack of money, in this statistical model, did not present a significant p-value in any of the vaccination periods.

**Table 2.** Estimation of logistic regression parameters applied to variables related to vaccination incompleteness of residents in health regions of Sergipe.

	Logistic OR (95%CI)	Corrected RR (95%CI)	p-value
0–12 months*			
Mother’s education (illiterate/primary (initial years) /incomplete elementary (final years)	1.22 (0.61–2.62)	1.17 (0.66–1.67)	0.586
Mother’s education (complete elementary school/incomplete secondary school)	0.86 (0.41–1.93)	0.88 (0.46–1.00)	0.710
Mother’s education (complete high school/incomplete higher education)	0.71 (0.34–1.58)	0.75 (0.39–1.00)	0.382
Vaccines are bad	1.36 (0.45–3.71)	1.26 (0.51–2.11)	0.567
Fear of a reaction	1.56 (1.07–2.25)	1.42 (1.06–1.69)	0.019
Friend or relative advised vaccination	0.72 (0.39–1.26)	0.77 (0.45–1.00)	0.268
No transport	1.12 (0.47–2.49)	1.09 (0.53–1.66)	0.788
No money	2.35 (0.98–5.72)	1.85 (0.98–2.78)	0.056
13–24 months^†^			
Mother’s education (illiterate/primary (initial years) /incomplete elementary (final years)	1.32 (0.63–2.96)	1.24 (0.68–1.65)	0.478
Mother’s education (complete elementary school/incomplete secondary school)	0.69 (0.30–1.63)	0.74 (0.35–1.00)	0.375
Mother’s education (complete high school/incomplete higher education)	0.83 (0.38–1.91)	0.86 (0.44–1.00)	0.639
Vaccines are bad	2.57 (0.89–7.38)	1.87 (0.91–2.88)	0.075
Fear of a reaction	1.52 (1.04–2.23)	1.37 (1.03–1.65)	0.032
Friend or relative advised vaccination	0.42 (0.21–0.79)	0.49 (0.26–0.00)	0.011
No transport	0.70 (0.26–1.71)	0.75 (0.32–1.00)	0.451
No money			
General (0–24 months)‡	2.51 (0.97–6.70)	1.85 (0.98–2.76)	0.059
Mother’s education (illiterate/primary (initial years) /incomplete elementary (final years)	1.13 (0.58–2.28)	1.09 (0.67–1.52)	0.717
Mother’s education (complete elementary school/incomplete secondary school)	0.85 (0.41–1.77)	0.89 (0.51–1.00)	0.649
Mother’s education (complete high school/incomplete higher education)	0.79 (0.40–1.63)	0.85 (0.49–1.00)	0.519
Vaccines are bad	1.17 (0.40–3.30)	1.11 (0.50–1.78)	0.762
Fear of a reaction	1.51 (1.07–2.13)	1.31 (1.05–1.53)	0.020
Friend or relative advised vaccination	0.57 (0.32–0.95)	0.67 (0.42–0.00)	0.037
No transport	0.81 (0.34–1.80)	0.87 (0.44–1.00)	0.614
No money	2.32 (0.97–5.73)	1.61 (0.98–2.15)	0.061

OR: odds ratio; RR: relative risk; CI: confidence interval.

*AUC: 0.61; Hosmer-Lemeshow Test=0.810

^†^ AUC: 0.63; Hosmer-Lemeshow Test=0.821

^‡^ AUC: 0.61; Hosmer-Lemeshow Test=0.893.

The variables fear of a reaction presented significant p-values for the first year (p-value of 0.019), for the second (p-value of 0.032) and for the first two years together (zero to 24 months) (p-value of 0.020) (Table 2).

As for the variable friend or relative advised vaccination, the p-values were significant in some cases, such as for the second year (p-value of 0.011) and considering the whole period (zero to 24 months) (0.037). For the period of the first year of life alone, the p-value was not significant (0.268) (Table 2).

## DISCUSSION

Considering the vaccination coverage recommended by the Ministry of Health’s vaccination procedure standards (2024), the BCG vaccine reached the target in all regions; the rotavirus vaccine reached the target only for the first dose and only in the health regions of Propriá, Glória and Estância; the pentavalent and VIP reached the target for the 1^st^, 2^nd^ and 3^rd^ doses in the regions of Propriá, Itabaiana and Lagarto; the pneumococcal vaccine did not meet the target for at any dose in any region; the meningococcal vaccine did not meet the target only in Glória, for the booster dose; for the triple viral, the first dose did not meet the target in any of the regions; DTP and VOP met the target only in the regions of Propriá and Lagarto; the triple viral, booster dose, failed to reach the recommended target only in Lagarto; the chickenpox vaccine did not meet the target in any of the cases; and the hepatitis A vaccine only met the target in Propriá, contributing to the argument that there is no homogeneity of results.^26,27^

Vaccines with a multiple-dose vaccination schedule usually have lower percentages in subsequent doses, as can be seen for the rotavirus, IPV, pneumococcal, and meningococcal vaccination schedules in all health regions and the pentavalent vaccination schedule in the regions of Estância, Itabaiana, Nossa Senhora da Glória, and Propriá. This trend of decreasing vaccination coverage is reported in other studies.^7^ The literature suggests that technological innovation applied to vaccine development reduces the need for multiple-dose schedules.^28,29^

Regarding the odds ratio associated with the percentages of vaccination incompleteness, it is possible to suggest that living in Propriá has a protective effect against vaccination incompleteness for vaccines recommended for the first two years of life (0–24 months), followed by Estância and Lagarto. Regarding the contributing factors, the regression analysis shows that the variable fear of a reaction was significant for 0–12, 13–24 and 0–24 months. In addition, having friends or relatives advise vaccination was significant for 13–24 months and 0–24 months.^30,31^

Despite the study’s limitations, such as: failure to include the yellow fever vaccine, which was not recommended in the local vaccination schedule; illegible vaccination records in the vaccination booklets; temporality; lack of monitoring of the number of home visits to identify the percentage of refusals to participate in the study; failure to include data from children living in the Aracaju and Nossa Senhora do Socorro health regions; lack of financial/logistical support; and the fact that, in addition to the tight schedule, more data were collected than the minimum sample size expected in the other health regions. Nevertheless, the methodology used in this study contributes to the reliability of the results with on-site collection, thus reducing the chances of underreporting or overreporting errors.^7-24,26-31^

It can be concluded that some of the results presented align with findings in the literature, such as heterogeneity in vaccination coverage percentages and a decline in vaccination coverage in subsequent doses in multi-dose vaccination schedules.^7,8,9,10,11,12,13,14,15,16,17,18,19,20,21,22,23,24,26,27,28,29,30,31^

Other results are unprecedented, such as the percentages of vaccination incompleteness by health regions and the odds ratios for vaccination incompleteness in regions of the state. In any case, the results are significant and innovative, providing extremely important evidence regarding the vaccination situation in recent years and contributing to the work of researchers and public managers in formulating improvements for public policies.

## Data Availability

The database that originated the article is available with the corresponding author.
